# Rapidly Reversible Monitored Anesthesia Care with Remimazolam and Flumazenil Reversal for Transcatheter Aortic Valve Replacement: A Randomized Controlled Trial

**DOI:** 10.3390/jcm15207880

**Published:** 2026-10-13

**Authors:** Atsuhiro Kitaura, Takashi Mino, Yukari Yoshino, Hiroatsu Sakamoto, Mayuka Matsushima, Yoshinobu Nakayama, Yasufumi Nakajima

**Affiliations:** 1 Department of Anesthesiology, Faculty of Medicine, Kindai University, Sakai 580-0197, Osaka, Japan; takashi.mino@med.kindai.ac.jp (T.M.); ynakayama@med.kindai.ac.jp (Y.N.);; 2 Department of Anesthesiology, Nagoya University, Nagoya 466-8550, Aichi, Japan; yoshino.yukari.a1@f.mail.nagoya-u.ac.jp; 3 Department of Anesthesiology, Center for Outcomes Research, University of Texas Health Science Center, Houston, TX 77030, USA

**Keywords:** remimazolam, monitored anesthesia care, transcatheter aortic valve replacement, dexmedetomidine

## Abstract

**Background/Objectives:** Monitored anesthesia care (MAC) is increasingly used for transcatheter aortic valve replacement (TAVR), where rapid recovery of consciousness may facilitate early postoperative neurological assessment. Remimazolam provides sedation that can be pharmacologically reversed with flumazenil. We compared emergence from sedation between a remimazolam–flumazenil-based MAC strategy and a conventional dexmedetomidine-based strategy in patients undergoing transfemoral TAVR. **Methods:** In this single-center randomized controlled trial, 84 patients undergoing transfemoral TAVR were randomized 1:1 to remimazolam–remifentanil (R group) or dexmedetomidine–propofol–remifentanil (D group) MAC. Flumazenil was administered at the initiation of the postoperative awakening attempt in the R group. The primary outcome was arousal time, assessed using two prespecified measures: time from discontinuation of all anesthetic infusions to arousal and time from initiation of the postoperative awakening attempt to arousal. Arousal was defined as a Modified Observer’s Assessment of Alertness/Sedation score of 5. **Results:** Eighty-three patients were included in the analysis (R group, *n* = 42; D group, *n* = 41). The time from discontinuation of all anesthetic infusions to arousal was shorter in the R group than in the D group (19.05 [15.77–23.59] vs. 24.09 [19.25–34.78] min; *p* = 0.0045). The time from initiation of the awakening attempt to arousal was also shorter (0.60 [0.33–1.00] vs. 1.00 [0–4.72] min; *p* = 0.0117). No patient in the R group required rescue propofol. No significant between-group differences were observed in major procedural or postoperative outcomes. **Conclusions:** A remimazolam-based MAC strategy with flumazenil reversal provided faster and more reliable emergence from sedation than a conventional dexmedetomidine-based strategy during transfemoral TAVR. These findings suggest that a reversible sedation strategy may facilitate rapid recovery of consciousness when early postoperative assessment is desired, although its impact on neurological or other clinical outcomes remains to be established.

## 1. Introduction

Transcatheter aortic valve replacement (TAVR) has become an established treatment for symptomatic severe aortic stenosis, particularly in older patients and those with multiple comorbidities. Although less invasive than surgical aortic valve replacement, TAVR is associated with substantial hemodynamic perturbations and a risk of neurological complications, including cerebral embolism [1,2,3,4,5]. Accordingly, anesthetic management for TAVR should provide adequate procedural sedation and hemodynamic stability while allowing prompt recovery of consciousness to facilitate early postoperative neurological assessment [6].

Monitored anesthesia care (MAC) is increasingly used for transfemoral TAVR and has been associated with reduced vasopressor and fluid requirements and shorter hospital stays compared with general anesthesia [7,8,9]. Dexmedetomidine is commonly used for MAC because it provides sedation with relatively limited respiratory depression [10]. However, adequate sedation during TAVR may require adjunctive sedatives such as propofol, particularly during highly stimulating procedural steps [11]. Moreover, the relatively prolonged sedative effect of dexmedetomidine and the lack of a specific reversal agent may make emergence less predictable. This may be clinically relevant after TAVR, where residual sedation can interfere with the immediate assessment of consciousness and neurological function.

Remimazolam is an ultra-short-acting benzodiazepine characterized by rapid metabolism, limited accumulation, and relatively modest cardiovascular depression [12,13,14,15,16]. Importantly, its sedative effects can be rapidly reversed with flumazenil. These pharmacological properties make a remimazolam–flumazenil-based strategy potentially attractive for MAC during TAVR, where both hemodynamic stability and rapid, predictable emergence are desirable. In our previous retrospective study, remimazolam-based MAC was feasible for TAVR and was associated with shorter awakening times than a dexmedetomidine-based approach [11]. However, the retrospective design was susceptible to treatment-selection bias and did not allow systematic evaluation of a prospectively defined remimazolam–flumazenil reversal strategy or postoperative recovery. Furthermore, prospective evidence regarding remimazolam-based MAC for TAVR remains limited [11,17,18,19,20,21].

Therefore, we conducted a randomized controlled trial to determine whether faster emergence could be reproduced under randomized allocation and to evaluate the controllability of emergence with a remimazolam–flumazenil-based MAC strategy compared with a conventional dexmedetomidine–propofol-based strategy in patients undergoing transfemoral TAVR. Emergence was assessed using two prespecified arousal measures with blinded assessment. Postoperative recovery and cognitive outcomes were also prospectively evaluated.

## 2. Materials and Methods

**Figure 1:**
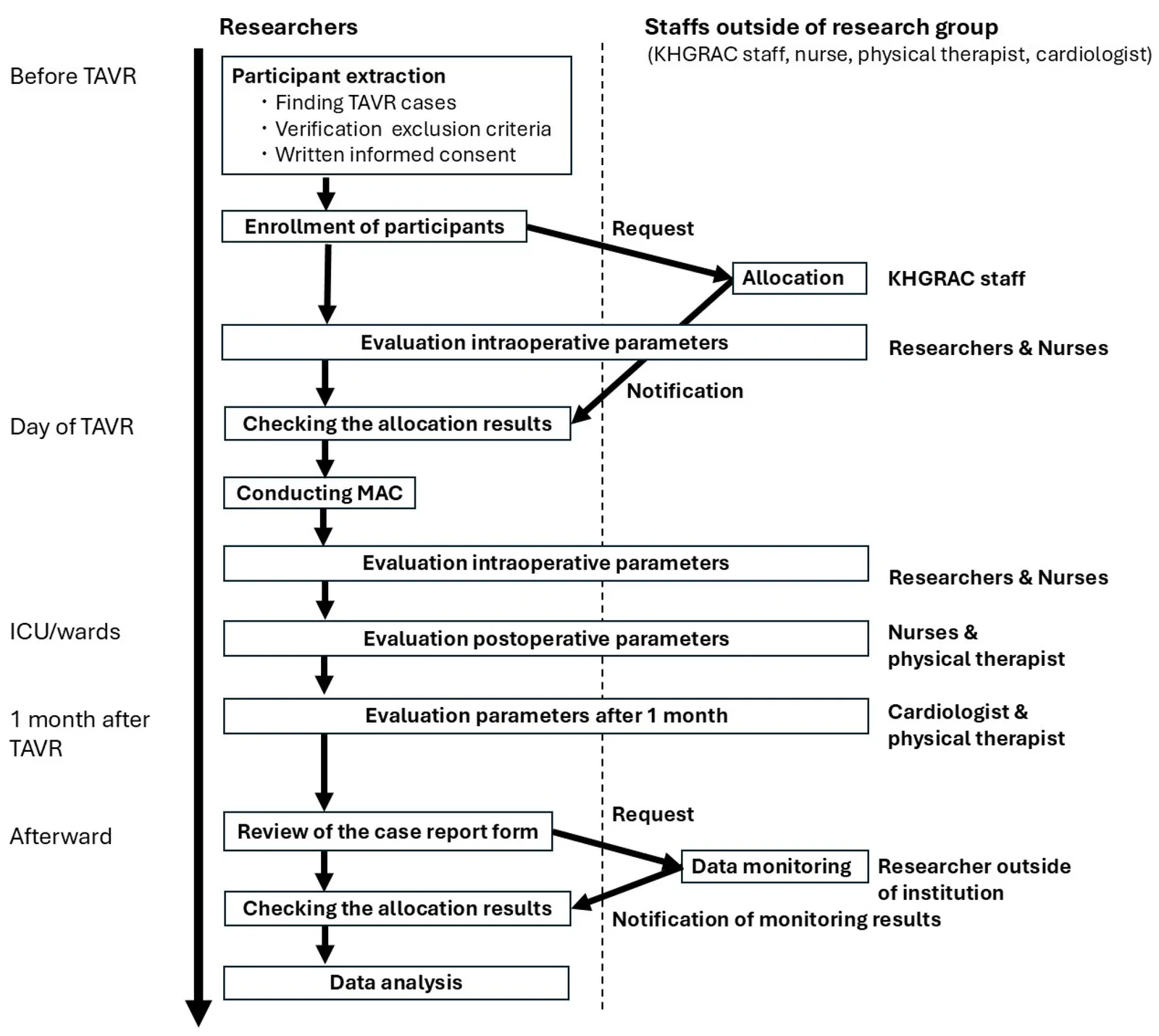
Research plan for this study. The objectivity of the study was ensured by involving non-researcher staff in the assignment and evaluation of the assessment data. KHGRAC, Kindai Hospital Global Research Alliance Center (Research Support Organization of the author’s institution); ICU, intensive care unit; MAC, monitored anesthesia care; TAVR, transcatheter aortic valve replacement.

This single-center randomized controlled trial was approved by the Institutional Review Board of Kindai University Faculty of Medicine (No. R05-136; 25 December 2023) and prospectively registered with the UMIN Clinical Trials Registry (UMIN-CTR; registration No. R000061952). Written informed consent was obtained from all participants. An overview of the study design is presented in Figure 1. This study was reported in accordance with the CONSORT guidelines (Supplementary Materials). 

### 2.1. Participants

Adult patients aged ≥18 years with severe aortic stenosis who were scheduled to undergo transfemoral transcatheter aortic valve replacement (TF-TAVR) under monitored anesthesia care (MAC) at our institution between 29 April 2024 and 20 December 2024 were prospectively screened for eligibility. Patients were excluded if they were receiving sedatives or opioids preoperatively, required preoperative tracheal intubation or mechanical ventilation, or required preoperative mechanical circulatory support.

All patients scheduled for TF-TAVR during the study period were screened using electronic medical records and preoperative assessments. Eligible patients received a written explanation of the study before the day of surgery, and those who provided written informed consent were enrolled. Participants were assigned sequential case numbers in the order of enrollment. No compensation was provided for study participation.

### 2.2. Group Assignment

Participants were randomized in a 1:1 ratio to one of two groups: (1) the R group, in which TAVR was performed under MAC using a remimazolam–remifentanil protocol, or (2) the D group, in which TAVR was performed under MAC using a dexmedetomidine–propofol–remifentanil protocol. Randomization was performed using a minimization method with a random component, with age (≥75 or <75 years) and sex as allocation factors.

To ensure allocation concealment and independence from the investigators, randomization was performed by an independent research support organization at our institution (Kindai Hospital Global Research Alliance Center [KHGRAC]) using the INDICE Cloud case registration and allocation system (UMI Medical Research Support System Cloud version). The investigators provided the KHGRAC allocation staff only with the participant’s case number, age, and sex. The allocation staff performed randomization using INDICE Cloud and informed the attending anesthesiologist of the assigned group on the day of TAVR. The investigators had no access to the allocation process before assignment.

### 2.3. MAC Protocols

MAC was performed in all participants according to our institutional protocol. In accordance with our institutional routine clinical practice, no premedication was administered. Angiotensin-converting enzyme inhibitors, angiotensin II receptor blockers, angiotensin receptor–neprilysin inhibitors, and oral antidiabetic agents were withheld on the day of TAVR. After positioning on the operating table, all participants underwent electrocardiography, pulse oximetry, noninvasive blood pressure monitoring, and bispectral index (BIS) monitoring (Aspect Medical Systems, Norwood, MA, USA). An arterial catheter was inserted under local anesthesia before induction, and arterial blood gas analysis was performed. The anesthetic protocol was determined according to group allocation.

In the R group, remimazolam was administered at a dose of 0.1 mg/kg for induction, followed by a continuous infusion at 1 mg/kg/h after loss of consciousness. Propofol (0.4 mg/kg per dose) was administered as rescue sedation if patient movement interfered with the procedure or clear signs of awakening were observed. In the D group, dexmedetomidine was administered at a loading dose of 4 μg/kg/h for 10 min, followed by a maintenance infusion at 0.7 μg/kg/h. Propofol (0.4 mg/kg per dose) was administered as needed during highly stimulating procedural steps, in response to patient movement interfering with the procedure, or when signs of awakening were observed. The decision to administer propofol was made by the attending anesthesiologist according to these predefined clinical criteria.

After loss of consciousness, a central venous catheter was inserted into the right internal jugular vein under ultrasound guidance with local anesthesia. Transthoracic echocardiography was used for intraoperative cardiac monitoring; transesophageal echocardiography was not routinely used. Spontaneous breathing was maintained throughout MAC, and upper airway patency was maintained using a jaw-elevation device (Hypnoz, San Diego, CA, USA). Remifentanil was continuously infused at 0.03 μg/kg/min in both groups, and the infusion rate was adjusted to maintain a spontaneous respiratory rate of 10–20 breaths/min. Acetaminophen (15 mg/kg) was administered intravenously for postoperative analgesia in all participants. After femoral arterial cannulation, heparin (100 U/kg) was administered to maintain an activated clotting time of ≥250 s. Dexamethasone sodium phosphate (6.6 mg) and granisetron (1 mg) were administered intravenously to all participants for prophylaxis against postoperative nausea and vomiting. BIS values were maintained between 40 and 70. Norepinephrine was administered when the mean arterial pressure decreased below 60 mmHg.

**Figure 2:**
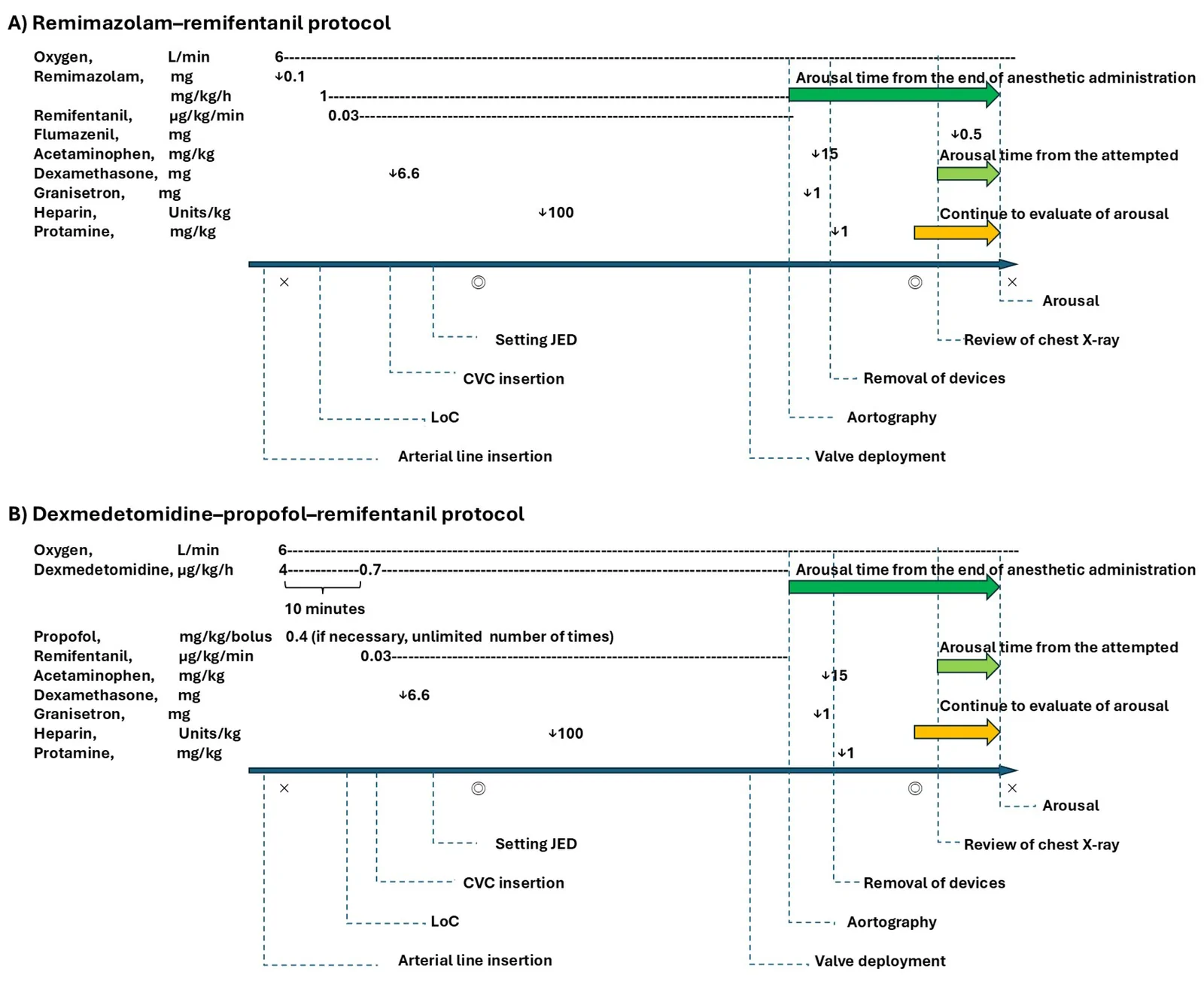
The MAC protocols: (**A**) The MAC protocol of the remimazolam group. (**B**) The MAC protocol of the dexmedetomidine group. CVC, central venous catheter; JED, jaw elevation device (exterior style of airway management device); LoC, loss of consciousness.

All anesthetic infusions were discontinued immediately after completion of TAVR valve deployment, and the time from discontinuation of all anesthetic infusions to arousal was recorded. The timing of anesthetic discontinuation was based on the institutionally optimized timing established in our pre-existing MAC protocol using dexmedetomidine, which was identical to the protocol used in the D group. After completion of the procedure and postoperative chest radiography, an active attempt to awaken the patient was initiated. Immediately after initiation of the awakening attempt, patients in the R group received 0.5 mg of intravenous flumazenil. Consciousness was assessed thereafter until arousal was confirmed. Patients who had not yet achieved arousal were transferred to the postanesthesia care unit (PACU), where assessment of consciousness was continued. The MAC protocol and sequence of awakening assessment are summarized in Figure 2.

### 2.4. Definition of Arousal Time

Arousal was defined as recovery to a Modified Observer’s Assessment of Alertness/Sedation (MOAA/S) score of 5. The primary outcome was arousal time, which was prospectively assessed using two prespecified measures: (1) the time from discontinuation of all anesthetic infusions after valve deployment to the first confirmed MOAA/S score of 5 and (2) the time from initiation of the postoperative awakening attempt to the first confirmed MOAA/S score of 5.

The postoperative awakening attempt was initiated after completion of chest radiography. The first MOAA/S assessment was performed immediately thereafter, and this time point was defined as 0 min for the second measure. Immediately after initiation of the awakening attempt, flumazenil (0.5 mg) was administered intravenously to patients in the R group. Consciousness was continuously assessed while the patient remained in the operating room. After transferring to the PACU, MOAA/S assessments were repeated every 5 min until a score of 5 was confirmed. The start time and the time at which an MOAA/S score of 5 was first confirmed were recorded in the anesthesia record.

### 2.5. Data Monitoring

To ensure the neutrality of the study, data monitoring was conducted by a research professional outside the research institution. Monitoring was performed three times: in the first case, the 50th case, and at the end of the study. The monitoring process included the following items: (1) obtaining written informed consent from the study participants, (2) confirming that the study participants met the eligibility criteria, (3) ensuring that there were no discrepancies between the case report forms and original documents or other study-related records, (4) verifying that data entry was performed according to the content of the case report forms, and (5) ensuring that the principal investigator conducted the study in compliance with relevant regulations and the study protocol. No issues requiring correction were reported during the three monitoring sessions.

### 2.6. Blinding

Attending anesthesiologists could not be blinded to group allocation because they administered the assigned anesthetic protocol. Nurses involved in the measurement of arousal time and assessment of the MOAA/S score were blinded to group allocation. Flumazenil administration was physically concealed from the nurses performing the arousal assessments so that they remained unaware of group allocation. Postoperative outcomes were assessed, as applicable, by healthcare professionals who were blinded to group allocation.

### 2.7. Measurements and Outcomes

The primary outcome was arousal time, assessed using two prespecified measures: (1) the time from discontinuation of all anesthetic infusions to arousal and (2) the time from initiation of the postoperative awakening attempt to arousal. These two measures were intended to jointly characterize complementary aspects of emergence: overall emergence from the anesthetic strategy and the controllability of awakening when recovery of consciousness was clinically required. Arousal was defined as the first confirmed MOAA/S score of 5, as described above. Although a formal criterion for overall primary-outcome success was not explicitly prespecified in the protocol, both measures were considered necessary to support the primary finding.

Secondary outcomes included sedation-related, hemodynamic, respiratory, procedural, and postoperative outcomes. Sedation-related outcomes included intraoperative patient movement, intraoperative awareness, airway complications, and propofol and remifentanil requirements. Hemodynamic outcomes included changes in mean arterial pressure and heart rate from the initiation of sedation to loss of consciousness, as well as intraoperative norepinephrine use. Respiratory outcomes included the minimum arterial pH and maximum PaCO_2_ during MAC. Procedural outcomes included anesthesia time, procedure time, TAVR procedural success, TAVR-related complications, postoperative aortic valve area, severity of aortic regurgitation, and new permanent pacemaker implantation. Postoperative outcomes included the incidence of postoperative nausea and vomiting (PONV), postoperative delirium, changes in cognitive function assessed using the Hasegawa Dementia Scale, quality of recovery assessed using the 15-item Quality of Recovery score (QoR-15), ICU length of stay, hospital length of stay, and 1-month survival.

Intraoperative outcomes, including the primary outcome, were assessed with the involvement of nurses who were blinded to group allocation. Attending anesthesiologists were aware of group allocation because they administered the assigned anesthetic protocol. To enhance the objectivity of arousal assessment, measurement of arousal time and assessment of the MOAA/S score were performed with the participation of blinded nurses. Postoperative outcomes were assessed by healthcare professionals, including nurses, intensive care physicians, and cardiologists, who were blinded to group allocation, as applicable. Baseline patient characteristics were obtained from the electronic medical records.

### 2.8. Statistical Analysis

#### Sample Size Calculation

The sample size was estimated based on data from our previous retrospective study [11]. In the pre-matching cohort of that study, the observed between-group difference in arousal time from discontinuation of anesthetic administration to arousal was 31.0 min, with a standard deviation of 43.8 min. Assuming a two-sided significance level of 0.05 and a statistical power of 80%, 32 participants per group were required based on a two-sample parametric comparison. Because arousal time was expected to have a non-normal distribution, group comparisons were planned to use the Wilcoxon rank-sum test. To account for the lower asymptotic efficiency of the Wilcoxon rank-sum test relative to the two-sample *t*-test, the estimated sample size was increased by 15% [22], resulting in 37 participants per group. Allowing for potential dropouts, five additional participants were included per group, resulting in a target sample size of 42 participants per group (84 participants in total).

### 2.9. Data Analysis

The primary outcome was arousal time, assessed using two prespecified measures: (1) the time from discontinuation of all anesthetic infusions to arousal and (2) the time from initiation of the postoperative awakening attempt to arousal. Both measures were compared between groups using the Wilcoxon rank-sum test, and two-sided *p*-values were calculated for each measure. No adjustment for multiplicity was applied to these two prespecified measures. To assess the potential impact of the post-randomization exclusion, a conservative sensitivity analysis was performed for the primary outcome. In this analysis, the excluded participant in the D group was assigned an arousal time of 0 min, representing an extreme assumption favoring the D group, and the between-group comparison was repeated using the Wilcoxon rank-sum test.

Continuous secondary outcomes were summarized by group and compared using the Wilcoxon rank-sum test. Categorical variables were compared using Fisher’s exact test or Pearson’s chi-square test, as appropriate. For categorical outcomes, between-group differences in proportions and their 95% confidence intervals (CIs) were also calculated where applicable. The 1-month survival rate was summarized by group, and the between-group difference and its 95% CI were calculated.

Analyses of secondary outcomes were considered exploratory. No adjustment for multiple comparisons was applied, and findings from secondary outcome analyses were interpreted accordingly.

The primary analysis was conducted on participants who received the allocated MAC intervention and for whom the primary outcome could be assessed. Participants who did not receive the allocated MAC intervention and for whom the primary outcome could not be assessed were excluded from the primary analysis. No imputation was performed for missing data; analyses were conducted using available cases.

Baseline patient characteristics were summarized using descriptive statistics: continuous variables were summarized by group, and categorical variables were reported as counts and percentages. All statistical analyses were performed using EZR version 1.68, a graphical user interface for R [23]. Unless otherwise specified, statistical significance was defined as a two-sided *p* < 0.05.

## 3. Results

### 3.1. Patient Characteristics

**Figure 3:**
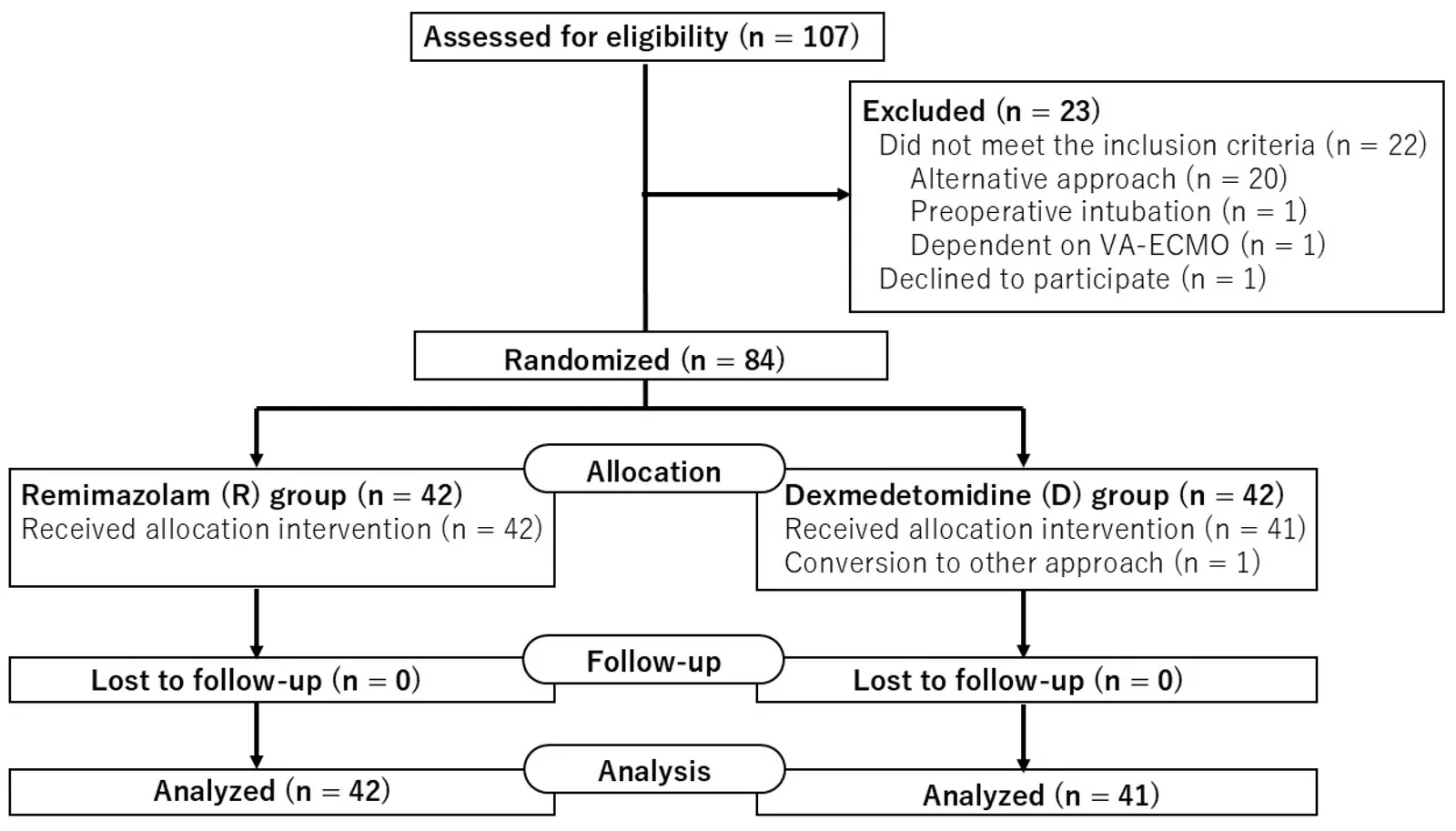
Flowchart of the study. VA-ECMO, veno-arterial extracorporeal membrane oxygenation.

A total of 107 patients were screened for eligibility. Of these, 85 were eligible for enrollment. One patient declined participation, and 84 participants were enrolled and randomized. After randomization, one patient assigned to the D group required conversion to trans-subclavian TAVR under general anesthesia and therefore did not receive the allocated MAC intervention. Because the primary outcome, arousal from the allocated MAC strategy, could not be assessed in this patient, the patient was excluded from the primary analysis. Consequently, 83 patients (42 in the R group and 41 in the D group) were included in the final analysis (Figure 3). All patients included in the analysis completed the prespecified follow-up.

Baseline patient characteristics are summarized in Table 1. Intraoperative and postoperative outcomes are presented in Table 2 and Table 3, respectively.

### 3.2. Primary Outcome

**Figure 4:**
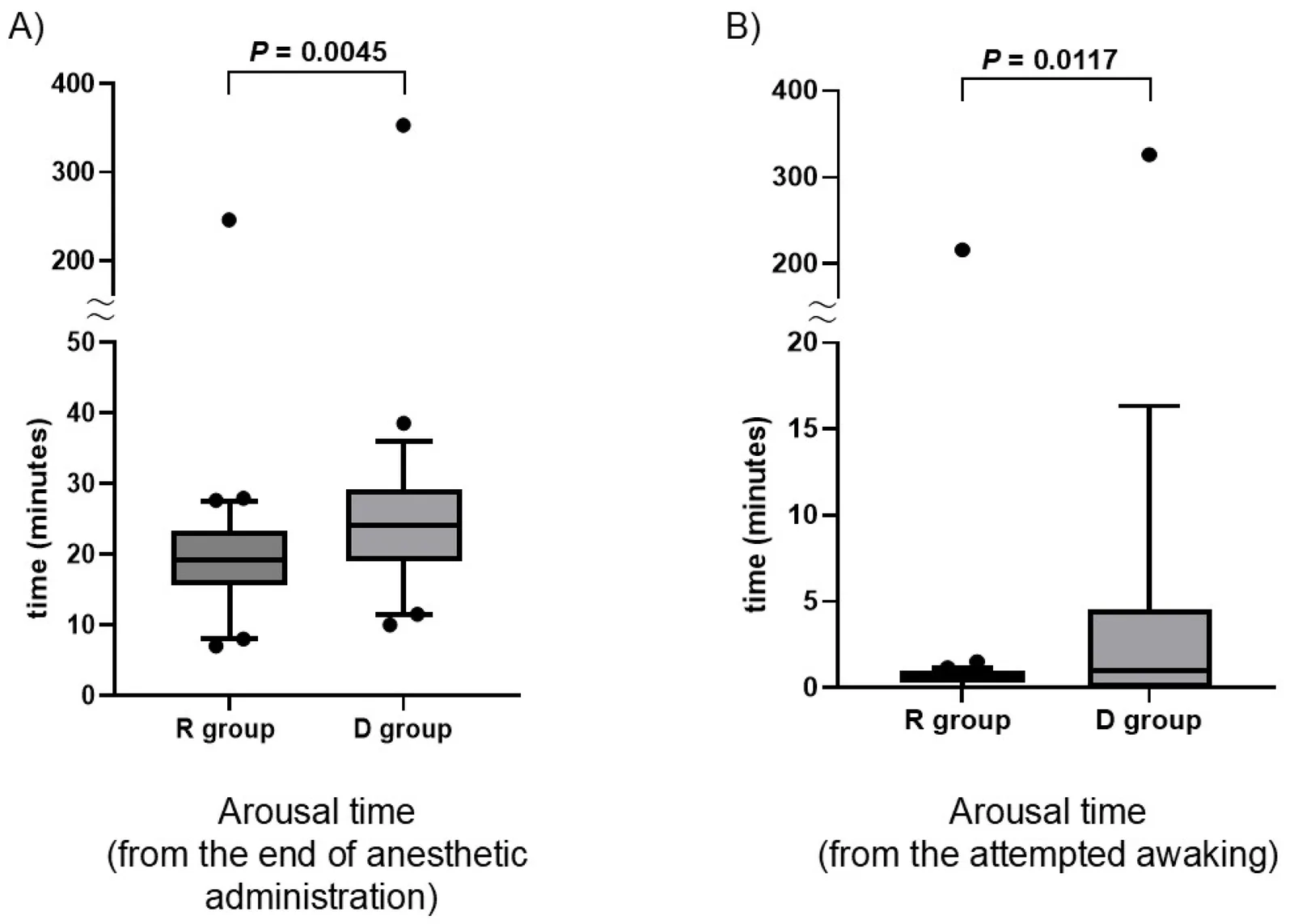
Arousal time: (**A**) Arousal time from discontinuation of all anesthetic infusions. (**B**) Arousal time from initiation of the postoperative awakening attempt. R group, remimazolam group; D group, dexmedetomidine group. Boxes indicate quartiles. Whiskers indicate the 5th and 95th percentiles.

The primary outcome, arousal time, was assessed using two prespecified measures. The time from discontinuation of all anesthetic infusions to arousal was significantly shorter in the R group than in the D group (19.05 [15.77–23.59] vs. 24.09 [19.25–34.78] min; Hodges–Lehmann location shift estimate [R minus D], −4.40 min [95% CI, −7.27 to −1.49], *p* = 0.0045). Similarly, the time from initiation of the postoperative awakening attempt to arousal was shorter in the R group (0.60 [0.33–1.00] vs. 1.00 [0–4.72] min; Hodges–Lehmann location shift estimate [R minus D], −0.51 min [95.07% CI, −1.98 to 0.00], *p* = 0.0117) (Figure 4). In the R group, arousal generally occurred within approximately 1 min after initiation of the awakening attempt, except in one patient who developed cerebral embolism. In the D group, five patients (12.2%) required >10 min to achieve arousal. In the conservative sensitivity analysis, in which the excluded participant in the D group was assigned an arousal time of 0 min, the between-group differences remained statistically significant for both measures of arousal time (time from discontinuation of all anesthetic infusions to arousal, *p* = 0.0094; time from initiation of the postoperative awakening attempt to arousal, *p* = 0.032).

### 3.3. Secondary Outcomes

Secondary outcomes are summarized in Table 2 and Table 3. No significant between-group differences were observed in anesthesia time (79.5 [70.25–94.25] vs. 82.0 [72.5–103.0] min; *p* = 0.3842) or procedure time (44.5 [37.0–54.5] vs. 49.0 [38.5–67.0] min; *p* = 0.2546). Remifentanil consumption also did not differ significantly between the groups (0.08585 [0.0717–0.1067] vs. 0.0958 [0.0805–0.115]; *p* = 0.1078). No patient in the R group required rescue propofol, whereas propofol was administered in the D group (0 [0–0] vs. 80 [50–100] mg; *p* < 0.001).

**Figure 5:**
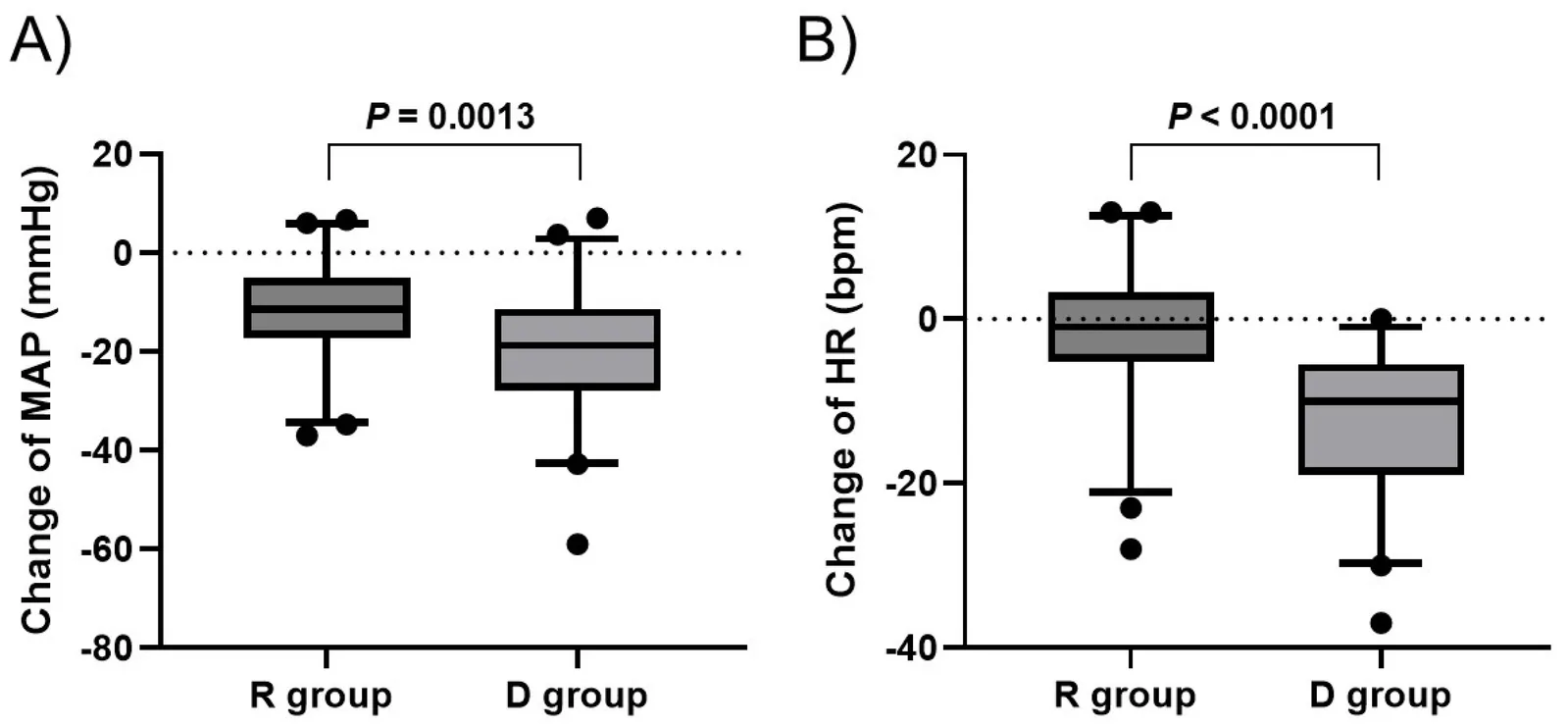
Changes in the circulatory indices during the induction of sedation: (**A**) Changes in the mean arterial pressure. (**B**) Changes in the heart rate. Changes in blood pressure and heart rate from the start of sedation (remimazolam or dexmedetomidine) to the time of loss of consciousness are shown. R group, remimazolam group; D group, dexmedetomidine group. Boxes indicate quartiles. Whiskers indicate the 5th and 95th percentiles. MAP, mean arterial pressure; HR, heart rate.

The decreases in mean arterial pressure and heart rate following anesthesia induction were greater in the D group than in the R group (mean arterial pressure: −11.50 [−16.91 to −5.66] vs. −18.66 [−27.66 to −11.66] mmHg; heart rate: −1 [−5.25 to 3.25] vs. −10 [−19 to −5.5] beats/min) (Figure 5). No significant between-group differences were observed in intraoperative patient movement, intraoperative awareness, airway complications, minimum arterial pH, maximum PaCO_2_, fluid administration, or the incidence or dose of norepinephrine use.

No significant between-group differences were observed in TAVR procedural success, effective orifice area, severity of aortic regurgitation, TAVR-related complications, or new permanent pacemaker implantation. Postoperative outcomes, including PONV, Hasegawa Dementia Scale scores, postoperative delirium, ICU length of stay, hospital length of stay, and 1-month survival, also showed no significant between-group differences. Because analyses of secondary outcomes were exploratory and were not adjusted for multiple comparisons, these findings should be interpreted accordingly.

## 4. Discussion

In this randomized controlled trial, a remimazolam-based monitored anesthesia care (MAC) strategy with flumazenil reversal resulted in significantly faster emergence than a conventional dexmedetomidine-based strategy in patients undergoing transfemoral transcatheter aortic valve replacement (TAVR). Importantly, these findings should not be interpreted simply as evidence of faster recovery with remimazolam itself. Rather, they reflect the characteristics of a reversible sedation strategy, in which sedation can be pharmacologically terminated with flumazenil when recovery of consciousness is clinically required.

Compared with our previous retrospective study, the present trial extends the evidence by evaluating the remimazolam–flumazenil strategy under randomized allocation, prospectively distinguishing overall emergence from the controllability of awakening, and systematically assessing postoperative recovery and cognitive outcomes.

The two prespecified measures of arousal time represent complementary aspects of emergence from sedation. The time from discontinuation of all anesthetic infusions to arousal reflects overall emergence from the anesthetic strategy, whereas the time from initiation of the postoperative awakening attempt to arousal reflects the practical controllability of emergence at the time when recovery of consciousness is clinically required. Both measures were shorter with the remimazolam–flumazenil strategy. In particular, the short interval between initiation of the awakening attempt and recovery of consciousness highlights the pharmacological reversibility provided by flumazenil rather than the intrinsic pharmacokinetic properties of remimazolam alone.

The relatively modest between-group difference in overall arousal time may partly be explained by the timing of anesthetic discontinuation in the present study. In both groups, anesthetic infusions were discontinued at a timing that had been previously optimized at our institution for the established dexmedetomidine-based MAC protocol, with the aim of facilitating timely emergence despite the relatively long context-sensitive half-time of dexmedetomidine. Consequently, the protocol was designed such that patients in the D group would often be approaching arousal by the time the postoperative awakening attempt was initiated, which may have allowed a proportion of patients in this group to awaken without substantial delay. From a methodological standpoint, a simpler design would have been to discontinue anesthetic administration at the completion of the procedure in both groups and compare the time from discontinuation to arousal. Such a design might have provided a more straightforward comparison of emergence between the two sedation strategies. However, we did not adopt this approach because deliberately continuing dexmedetomidine until the end of the procedure solely to standardize the study comparison could have unnecessarily prolonged sedation and subsequent emergence in patients assigned to the conventional dexmedetomidine-based strategy. We therefore prioritized a clinically established protocol rather than modifying the timing of anesthetic discontinuation solely for experimental standardization.

Although the between-group difference in overall arousal time was modest in absolute terms, its potential clinical relevance should be considered in the context of TAVR practice. At centers performing multiple TAVR procedures in a single day, even a difference of several minutes in recovery time per patient could accumulate across successive cases and potentially affect operating room workflow and resource utilization. However, the present study did not evaluate operating room turnover, staffing requirements, or procedural throughput, and therefore any operational benefit remains speculative.

From a clinical perspective, although TAVR is less invasive than surgical aortic valve replacement, cerebral embolic events remain an important procedural complication [1,2,3,4,5]. Once symptomatic, cerebral embolism can markedly impair a patient’s ability to perform activities of daily living, making it a highly concerning complication [24]. Residual sedation immediately after the procedure may complicate the neurological assessment of patients with delayed recovery of consciousness. Accordingly, having the option to actively facilitate awakening when needed may allow neurological status to be assessed earlier after the procedure. This may be clinically relevant because the benefit of reperfusion therapy for acute cerebral ischemia is time-dependent [25,26]. In the present study, most patients receiving the remimazolam–flumazenil strategy recovered consciousness rapidly after initiation of the awakening attempt, whereas several patients in the dexmedetomidine group required more than 10 min. However, the present study did not evaluate whether earlier recovery of consciousness resulted in earlier detection or treatment of neurological complications, nor was it designed or powered to assess neurological outcomes. Therefore, any potential neurological implications of rapid pharmacological reversal remain speculative and should be considered hypothesis-generating. Further studies are required to determine whether differences in the timing of recovery of consciousness have any measurable effect on the recognition, management, or outcomes of neurological complications.

The two groups also differed in their requirements for adjunctive sedation. In the dexmedetomidine group, propofol was administered as needed during highly stimulating procedural steps or when signs of inadequate sedation were observed. In contrast, no patient in the R group required rescue propofol, although rescue administration was permitted by the study protocol. A previous study comparing remimazolam- and dexmedetomidine-based MAC for TAVR, both in combination with remifentanil, similarly reported that requests from the proceduralist for deeper sedation were less frequent in the R group than in the dexmedetomidine group [27]. In the present study, all patients in the R group also completed TAVR under the assigned sedation protocol without requiring additional sedative agents. This finding suggests that a remimazolam-based sedation strategy could allow TAVR to be performed without additional sedatives, potentially reducing the need for adjunctive medications and simplifying anesthetic management. The observed difference in propofol use should be interpreted in the context of the prespecified differences between the two sedation protocols. Accordingly, the present findings should be interpreted as a comparison between two clinically applicable sedation strategies, rather than as a direct comparison of the pharmacological effects of remimazolam and dexmedetomidine.

Hemodynamic responses during anesthesia induction also differed between the groups. The decreases in mean arterial pressure and heart rate following induction were greater in the dexmedetomidine-based strategy than in the remimazolam-based strategy. These differences should be interpreted as reflecting the overall anesthetic protocols rather than the isolated pharmacological effects of remimazolam and dexmedetomidine. This finding may be clinically relevant in patients undergoing TAVR, who are often older and have limited cardiovascular reserves. However, no significant between-group differences were observed in the incidence or dose of intraoperative norepinephrine use. Moreover, the observed hemodynamic differences were limited to the induction period and should not be extrapolated to imply superior hemodynamic stability throughout the entire procedure. Because these hemodynamic outcomes were secondary exploratory outcomes, these findings should be interpreted cautiously.

No significant between-group differences were observed in procedural success, TAVR-related complications, postoperative recovery indices, ICU or hospital length of stay, or 1-month survival. Similarly, no significant differences were observed in postoperative delirium or cognitive function. Although benzodiazepine exposure has traditionally raised concerns regarding postoperative delirium, the present study was not powered to detect differences in relatively infrequent clinical outcomes. Accordingly, the absence of statistically significant differences should not be interpreted as evidence of equivalence or noninferiority between the two strategies with respect to safety or postoperative outcomes.

This study has several limitations. First, the intervention evaluated in the R group was a combined remimazolam–flumazenil strategy; therefore, the shorter arousal time cannot be attributed solely to the pharmacokinetic properties of remimazolam. In particular, the rapid emergence after initiation of the awakening attempt directly reflects, at least in part, pharmacological reversal with flumazenil. Because the study did not include an R group without flumazenil reversal, the independent contributions of remimazolam and flumazenil to the observed difference in arousal time cannot be disentangled. A three-arm study including remimazolam without flumazenil would be required to distinguish these effects. Second, the anesthetic regimens were not pharmacologically equivalent between the groups. Propofol was incorporated as an adjunct in the dexmedetomidine-based protocol and was administered according to predefined clinical criteria at the discretion of the attending anesthesiologist, whereas no patient in the R group ultimately required rescue propofol. These differences may have influenced both emergence and hemodynamic outcomes. Third, attending anesthesiologists could not be blinded to group allocation because they were required to administer the assigned anesthetic protocol. Although blinded nurses participated in the assessment of arousal and flumazenil administration was concealed from these assessors, knowledge of group allocation by the attending anesthesiologist may have influenced aspects of intraoperative management. Fourth, the present study was powered to detect differences in arousal time, an intermediate process-related endpoint, rather than major clinical or patient-centered outcomes. Therefore, although the remimazolam–flumazenil strategy enabled more rapid and controllable emergence, the present findings do not establish whether this characteristic translates into meaningful improvements in neurological outcomes, postoperative recovery, survival, or healthcare resource utilization. Fifth, although the two measures of arousal time were prespecified, no adjustment for multiplicity was applied to the primary outcome analyses. In addition, no adjustment for multiple comparisons was applied to the numerous secondary outcome analyses, increasing the possibility of type I error; therefore, statistically significant secondary findings should be considered exploratory. Sixth, one participant randomized to the D group did not receive the allocated intervention because the procedure was converted to trans-subclavian TAVR under general anesthesia and was therefore excluded from the primary analysis. Thus, the primary analysis did not strictly follow the intention-to-treat principle. However, a conservative sensitivity analysis assigning an arousal time of 0 min to this participant, thereby favoring the D group, did not materially alter the primary finding, suggesting that the post-randomization exclusion was unlikely to have substantially influenced the observed between-group difference. Finally, this was a single-center study with a relatively limited sample size, which limits the generalizability of the findings and the ability to detect differences in uncommon clinical outcomes.

Future multicenter studies with larger sample sizes should evaluate whether the ability to pharmacologically terminate sedation and facilitate rapid recovery of consciousness leads to earlier recognition of neurological abnormalities, a lower incidence of postoperative delirium, shorter recovery unit stays, improved patient-reported recovery, or reduced healthcare resource utilization. Such studies would help determine whether the more rapid and controllable emergence observed with this reversible sedation strategy ultimately translates into measurable patient-centered or operational benefits.

## 5. Conclusions

A remimazolam-based MAC strategy incorporating flumazenil reversal resulted in faster emergence from sedation than a conventional dexmedetomidine-based MAC strategy in patients undergoing transfemoral TAVR. These findings reflect differences between the complete anesthetic strategies and should not be attributed to the effects of the sedative agents alone. The reversible nature of the remimazolam–flumazenil strategy may facilitate rapid recovery of consciousness when early postoperative assessment is desired; however, whether this pharmacological controllability translates into improved neurological or other patient-centered outcomes remains to be established.

## Figures and Tables

**Table 1:** Patient characteristics.

	Remimazolam Group	Dexmedetomidine Group	*p*-Value
Age, years, median (IQR)	83 (80–87)	83 (79.5–86)	0.92 *
Male, n (%)	12 (28.5)	12 (29.2)	0.94 *
Height, cm, median (IQR)	150 (146–157)	150 (147–158)	0.45 *
Body weight, kg, median (IQR)	50 (44.375–55.875)	50 (45–58.7)	0.51 *
Body mass index, kg/m^2^, median (IQR)	22.417 (20.745–24.664)	21.303 (19.848–24.232)	0.49 *
ASA-physical status	4	4	N/A
EuroSCORE II, %, median (IQR)	2.85 (1.777–4.075)	2.96 (1.845–3.77)	0.90 *
NYHA classification			0.32 **
1, n (%)	0 (0)	0 (0)	
2, n (%)	37 (88.1)	35 (85.4)	
3, n (%)	2 (4.8)	5 (12.2)	
4, n (%)	3 (7.1)	1 (2.4)	
Clinical Frailty Scale			0.13 *
1, n (%)	2 (4.7)	0 (0.0)	
2, n (%)	8 (19.0)	3 (7.3)	
3, n (%)	6 (14.2)	12 (29.2)	
4, n (%)	16 (38.1)	9 (21.9)	
5, n (%)	5 (11.9)	9 (21.9)	
6, n (%)	3 (7.1)	3 (7.3)	
7, n (%)	2 (4.7)	4 (9.7)	
8, n (%)	0 (0.0)	1 (2.4)	
Hasegawa’s Dementia Scale, points, median (IQR)	27.5 (21.25, 29)	27 (22, 29.25)	0.55 *
Medical history			
Hypertension, n (%)	38 (90.4)	39 (95.1)	0.41 ***
Diabetes, n (%)	9 (21.4)	9 (21.9)	0.95 ***
Ischemic heart disease, n (%)	9 (21.4)	7 (17.0)	0.61 ***
Hemoglobin, g/dL, median (IQR)	12.1 (10.925–12.825)	11.8 (10.05–12.65)	0.21 *
eGFR, mL/min/1.73 m^2^, median (IQR)	52.15 (41.9–64.65)	47.1 (33.45–65.1)	0.59 *
BNP, pg/mL, median (IQR)	207.25 (96.825–507.85)	150.2 (90.15–241.6)	0.30 *
Echocardiogram			
LVEF, %, median (IQR)	68 (57.5–72)	68 (60–73)	0.67 *
LVDd, mm, median (IQR)	42 (40–46.25)	43 (39–49)	0.51 *
AVA, cm^2^, median (IQR)	0.775 (0.625–0.94)	0.805 (0.71–0.94)	0.49 *
AV mPG, mmHg, median (IQR)	42 (35–53.5)	40 (34–45)	0.56 *
Medication			
Calcium channel blocker, n (%)	15 (35.7)	21 (51.2)	0.18 ***
Beta blocker, n (%)	13 (30.9)	12 (29.2)	0.86 ***
ACEI/ARB/ARNI, n (%)	19 (45.2)	17 (41.4)	0.27 ***
Apfel score, points, median (IQR)	2 (0–2)	2 (0–2)	0.63 *
Self-expanding device, n (%)	13 (30.95)	18 (43.90)	0.07 ***

* Wilcoxon rank-sum test, two-sided. ** Fisher’s exact test, two-sided. *** Pearson’s chi-square test, two-sided. ASA, American Society of Anesthesiologists; ACEI, angiotensin-converting enzyme inhibitors; ARB, angiotensin II receptor blocker; ARNI, angiotensin receptor neprilysin inhibitor; AVA, aortic valve area; AV mPG, mean pressure gradient across the aortic valve; BNP, brain natriuretic peptide; eGFR, estimated glomerular filtration rate; LVEF, left ventricular ejection fraction; LVDd, left ventricular end-diastolic diameter; NYHA, New York Heart Association.

**Table 2:** Intraoperative outcomes.

	Remimazolam Group	Dexmedetomidine Group	*p*-Value
Arousal time (from the end of anesthetic administration), minutes, median (IQR)	19.05 (15.77–23.59)	24.0915 (19.25–34.78)	0.0045 *
Arousal time (from the attempted awakening), minutes, median (IQR)	0.6 (0.33–1)	1 (0–4.72)	0.0117 *
Anesthesia time, minutes, median (IQR)	79.5 (70.25–94.25)	82 (72.5–103)	0.38 *
Operation time, minutes, median (IQR)	44.5 (37–54.5)	49 (38.5–67)	0.25 *
Infusion volume, mL, median (IQR)	1200 (850–1600)	1250 (1025–1600)	0.58 *
Intraoperative remifentanil dose, µg, median (IQR)	0.08585 (0.0717–0.1067)	0.0958 (0.0805–0.115)	0.10 *
Intraoperative propofol dose, mg, median (IQR)	0 (0–0)	80 (50–100)	<0.001 *
Intraoperative dexmedetomidine dose, mg, median (IQR)	0 (0–0)	0.0607 (0.0509–0.0684)	<0.001 *
Cases requiring noradrenaline, n (%)	32 (76.1%)	32 (78.0%)	0.79 **
Intraoperative noradrenaline dose, mg, median (IQR)	0.0662 (0.00645–0.181)	0.108 (0.01–0.298)	0.14 *
Intraoperative minimum value of pH, median (IQR)	7.317 (7.283–7.334)	7.323 (7.294–7.362)	0.20 *
Intraoperative maximum value of PaCO_2_ and Torr, median (IQR)	50.25 (45.85–54.77)	47.3 (44.25–52.25)	0.10 *
Intraoperative body movement with procedure interruption, times, median (IQR)	0 (0–0)	0 (0–0)	0.16 *
Intraoperative memory, n (%)	0 (0%)	0 (0%)	NA
Airway trouble, n (%)	0 (0%)	0 (0%)	NA
Changes in blood pressure during induction, mmHg, median (IQR)	−11.50 (−16.91–−5.66)	−18.66 (−27.66–−11.66)	0.0013 *
Changes in heart rate during induction, bpm, median (IQR)	−1 (−5.25–3.25)	−10 (−19–−5.5)	<0.0001 *
TAVR success rate, n (%)	42 (100)	41 (100)	NA
TAVR surgical complication, n (%)	0 (0%)	0 (0%)	NA
ECMO required, n (%)	0 (0%)	1 (2.4%)	0.13 ***
Intraoperative cerebral embolism	1 (2.3%)	0 (0%)	0.33 ***

*p*-value: * Wilcoxon rank-sum test, two-sided. ** Pearson’s chi-square test, two-sided. *** Fisher’s exact test, two-sided. TAVR, transcatheter aortic valve replacement; ECMO, extracorporeal membrane oxygenation.

**Table 3:** Postoperative outcomes.

	Remimazolam Group	Dexmedetomidine Group	*p* Value
Prognosis			
ICU stay, hours, median (IQR)	20.125 (16.625–23)	19 (7.5–22)	0.10 *
Hospital stay, days, median (IQR)	5 (4–6)	4 (4–5)	0.28 *
1-month survival rate, %	100	100	N/A
Status of the TAVR valve			
AVA, cm^2^, median (IQR)	1.97 (1.5–2.2025)	1.97 (1.775–2.29)	0.21 *
AR			0.33 **
None, n	16	10	
Trivial–mild, n	26	31	
>moderate, n	0	0	
Pacemaker implantation, n (%)	0 (0)	0 (0)	N/A
Quality of postoperative recovery			
QoR 15 score, points, median (IQR)	144.5 (140–149.5)	146 (140–150)	0.96 *
Postoperative nausea and vomiting, n (%)	3 (7.1)	0 (0.0)	0.24 *
Nausea score			0.24 **
0	39	41	
1	3	0	
Food intake on POD2, %, median (IQR)	90 (70–100)	100 (70–100)	0.81 *
Food intake on POD3, %, median (IQR)	100 (70–100)	100 (70–100)	0.43 *
Hasegawa’s Dementia Scale, points, median (IQR)	25 (22–27.75)	26 (19.25–29)	0.60 *
Change in Hasegawa’s Dementia Scale before and after TAVR, points, median (IQR)	−0.5 (−3.75–0.75)	−2 (−2–1)	0.90 *
Postoperative delirium, n (%)	1 (2.38%)	5 (12.0%)	0.10 **

* Wilcoxon rank-sum test, two-sided. ** Fisher’s exact test, two-sided.

## Data Availability

The data presented in this study are available from the corresponding author upon reasonable request, subject to applicable ethical and institutional requirements. The data are not publicly available due to privacy and ethical restrictions.

## Supplementary Materials

The following supporting information can be downloaded at https://www.mdpi.com/article/10.3390/jcm15207880/s1, The CONSORT 2025 checklist [28] is available as Supplementary Materials.
